# Emulsion Explosives: A Tutorial Review and Highlight of Recent Progress

**DOI:** 10.3390/ma15144952

**Published:** 2022-07-15

**Authors:** Bartlomiej Kramarczyk, Krystyna Suda, Patrycja Kowalik, Kuba Swiatek, Katarzyna Jaszcz, Tomasz Jarosz

**Affiliations:** 1 NITROERG S.A., 1 Alfred Nobel Square, 43-150 Bierun, Poland; b.kramarczyk@nitroerg.pl; 2 Faculty of Chemistry, Silesian University of Technology, 44-100 Gliwice, Poland; kryssud364@student.polsl.pl (K.S.); patrkow960@student.polsl.pl (P.K.); kubaswi454@student.polsl.pl (K.S.); katarzyna.jaszcz@polsl.pl (K.J.)

**Keywords:** emulsion explosive, energetic material, ammonium nitrate, detonation, ANFO

## Abstract

Emulsion explosives (EE) have been commercially available in various forms for over 50 years. Over this period, the popularity and production technology of this class of energetic materials have been developing constantly. Despite this rapid rise to prominence and, in some applications, prevalence over traditional energetic materials, remarkably little information is available on the physicochemical and energetic properties of these materials and factors affecting those properties. This work is dedicated to presenting the fundamental information relevant to the features, properties and applications of EEs, while highlighting the most significant recent progress pertaining to those materials. Particular emphasis has been given to providing information about the types, composition, modifications and detonation parameters of EEs, as well as to highlighting the less obvious, emerging applications of EEs.

## 1. Introduction

Emulsion explosives (EE) are one of the most recently developed classes of energetic materials [1,2,3,4,5] and can be perceived as a step forward from the traditional energetic materials based on ammonium nitrate (AN), such as amatols, ammonals and ANFO (ammonium nitrate-fuel oil). EEs are obtained by physically or chemically sensitising an “EE matrix”, which is a water-in-oil emulsion, containing at the very least ammonium nitrate, water, oils and a surfactant [6].

The unique feature of EE matrices is that they are insensitive to initiation and cannot sustain detonation without being sensitised. This feature resulted in them being classed as oxidising agents, specifically with no. UN 3375, being assigned to class 5.1 as per ADR/RID regulations [7]. Due to this classification, the requirements for transporting EE matrices are much less stringent than the requirements for transporting traditional energetic materials (which are assigned to ADR/RID class 1).

The facile transportation of EE matrices was the key factor underlying the development of bulk emulsion explosives. Bulk EEs, which are a novel form of energetic materials, can be produced directly within boreholes. This is achieved via loading the boreholes with a mixture of the EE matrix and a chemical sensitising agent. This approach eliminates the need for transporting and handling explosive materials prior to the blasting operation, while allowing remote loading of boreholes. The key advantages of bulk EEs in comparison with traditional cartridged energetic materials are increased safety and facile borehole loading.

**Figure 1:**
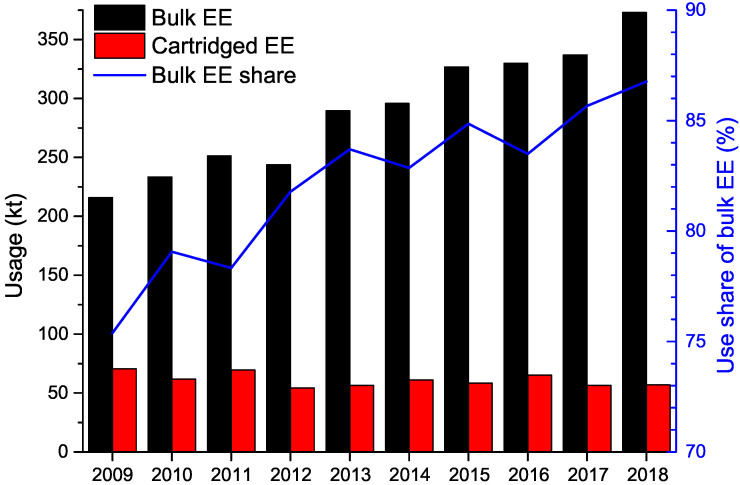
Usage of bulk and cartridged EEs and share of bulk EEs in the total usage of EEs in Europe (EU member states, Norway and Switzerland) over recent years. Data provided courtesy of the Federation of European Explosives Manufacturers.

Recent years have also brought about a significant improvement in the performance of bulk EEs [8], resulting in their increasingly popular use, particularly in comparison with cartridged EEs (Figure 1), as their usage share in the total usage of EEs has exceeded 85% in 2018 and is expected to have risen even further in the last several years. The primary application of EEs is in blasting operations oriented towards open pit and underground mining. The development and commercialisation of coal dust- and methane-permitted EEs has also contributed to area of application, allowing bulk EEs to compete with traditional energetic materials in this area as well. Considering the scale, on which bulk EEs are utilised annually, even seemingly minor, incremental improvements of their performance are expected to have significant economic impact and improvements to the safety parameters of bulk EEs are of even greater importance. Consequently, developing new EE formulations exhibiting improved properties is highly desirable from a number of viewpoints.

The rational development of EEs requires a thorough understanding of the processes occurring in the manufacture and handling of EE matrices, as well as those associated with chemically sensitising EEs. Despite the increasing popularity of EEs, however, no comprehensive source of the above information is available in the scientific and technical literature. The sole recent review of advances in the rheology of EEs [9] is a valuable work, but does not facilitate access to the subject as it focuses on a highly-specialised subject area. This work is supplemented by a more dated summary of the issues of the stability and rheology of EE matrices [10]. Even so, the available literature results in high entry requirements for the subject, translating into its limited accessibility, significantly hindering the development of EEs.

In light of the above, this tutorial review was intended to comprehensively collect the essential and fundamental information about various aspects of EEs for the first time, so as to facilitate access to the subject of EEs by non-specialists. The collated account of recent developments relevant to EEs can both serve as examples of current issues and means of highlighting the most significant developments in the subject area.

## 2. Stability of EE Matrices

Emulsions are thermodynamically unstable dispersion systems that are susceptible to aging via, e.g., Ostwald ripening, flocculation, creaming and coalescence, resulting in phase separation and a gradual deviation from the properties of the initial emulsion [11]. Due to the high volume fraction of the dispersed phase in the EE matrices, they are highly resistant to flocculation and creaming [12]. Instead, the most relevant mode of EE matrix aging is that of emulsion-to-suspension transition, due to the gradual crystallisation of ammonium nitrate [9]. Studies of this crystallisation process have revealed that it takes place in the EE matrix droplets oversaturated with ammonium nitrate, resulting in the formation of pure ammonium nitrate crystals and droplets of unsaturated ammonium nitrate solutions [13].

The choice of emulsifying agent used to produce the EE matrices is highly relevant to the stability of the EE formulation. In the case of poly(isobutylene)-succinic anhydride (PIBSA) modified with either diethanolamine or with poly(ethylene glycol) oligomers, the choice of emulsifying agent was found to have a significant impact on the stability and rheological properties of EE matrices [14]. Interestingly, even though two compounds exhibiting high performance (preventing phase separation and resulting in a minimal loss of ammonium nitrate from the EE matrix during aging studies) were identified, the hydrophile-lipophile balance of the emulsifying agents was found to have little correlation on their efficacy.

In a further study, a more diverse choice of emulsifying agents, as well as their mixtures has been tested [15], revealing that polymeric emulsifying agents were in general more effective in maintaining the EE matrix than low-molecular agents, despite them affording higher interfacial tension values than those afforded by low-molecular agents. Interestingly, among the tested emulsifying agents, polypropylene (denoted as H036) was found to be more effective than agents bearing various functional groups.

## 3. Approaches to Sensitising EEs

The production safety philosophy of EEs is that non-explosive components are mixed together to form an emulsion, which is referred to as the “EE matrix”. The matrix is not an explosive, as its density and homogeneity do not allow for supporting the detonation processes. To achieve the transition from this non-explosive state to an explosive state, in which the EE is capable of sustained detonation, the EE matrix must be sensitised. Sensitising consists of supplementing the matrix with substances that lower the density and introduce homogeneity “defects”, such as small gas-filled spheres, which constitute hot-spots. Upon detonation, the gas bubbles (hot-spots) absorb energy, heating up to high temperatures, allowing the detonation to be sustained. Physical and chemical sensitisation methods are employed, with the choice of method being strongly correlated with the type of EE (cartridged or bulk) being sensitised.

Physical sensitisation consists of supplementing the EE matrix with glass microspheres (tiny glass beads of very low density) and is the prevalent form of sensitisation for producing cartridged EEs. Chemical sensitisation, on the other hand, is mainly employed for bulk EEs. It relies on the reaction of the sensitising agent with oxidising agents constituting the EE matrix, as this reaction results in the evolution of gas that produces small bubbles across the entire volume of the sensitised matrix. In both cases, sensitisation results in the density of the EE matrix decreasing.

An important consideration for sensitised EEs is that of the optimal size and population of the low-density (microspheres or gas bubbles) defects—“pores” in the EE volume. It has been found that the diameter of the pores in sensitised EEs strongly impacts their detonation velocity and critical diameter [16]. Consequently, this aspect of sensitisation should also be taken into account during the development of EEs, with control over the dimensions and distribution of pores likely being the key aspects of developing new EE sensitisation methods. In the case of physical sensitisation, there is no problem with maintaining uniform pore size. Microspheres can readily be manufactured to have a narrow size distribution. In the case of chemical sensitisation, however, the size of the gas bubbles depends primarily on the type of sensitising agent, sensitisation time, temperature and EE matrix viscosity. Nitrogen released in the reaction of sodium nitrite with ammonium nitrate in the initial phase of sensitisation generates very small bubbles. as the reaction proceeds, they begin to increase in volume until all the sodium nitrite has been reacted. The bubbles should be as small as possible and perfectly distributed throughout the mass of the emulsion.

### 3.1. Physical Sensitising

Physical sensitisation relies on adding solid spheres that are filled with gas to the EE matrix. Glass and polymer microspheres are most commonly used for the purpose of physical sensitisation, but perlite is sometimes used as well [17,18]. The amount of sensitising agent in the final EE formulation is typically in the range of 1–4 wt.%, depending on the type of microspheres and the desired density of the final EE. Polymer microspheres are more effective in reducing density than glass ones [19]. It should be noted, however, that the comparison between the two types of microspheres was conducted based on the density of the sensitised EEs rather than on their weight share in the formulation and the nearly twofold difference in microsphere diameters is likely a highly significant factor. Despite this potential unresolved issue, polymer microspheres have a significant advantage in their facile processing, enabling their fabrication directly on-site. The use of perlite is currently declining, due to the rapidly developing technology for the production of glass microspheres.

In terms of the physical sensitisation of EEs, cenospheres appear to be a viable alternative to glass and polymer microspheres [20], as velocity of detonation (VoD) reported for cenosphere-based EEs are comparable to those reported for glass microsphere-based EEs. The added value of cenospheres in comparison with other types of physical sensitising agents is that they are a waste material and as such require no significant energy or material investments to be obtained.

### 3.2. Chemical Sensitising

Chemical sensitisation is mainly used for bulk EE. In some cases this type of sensitisation is also used for the cartridges. It involves a chemical reaction between the sensitising solution and the oxidising agent phase of the EE matrix. Aqueous solutions of sodium nitrite are primarily used as chemical sensitising agents. The use of sodium nitrite is based on a reaction between sodium nitrite and acidified ammonium nitrate in the presence of thiourea. This reaction produces nitrogen which, in the form of micro bubbles, fills the entire volume of the emulsion, reducing the density and creating hot spots. The rate of this reaction is strictly dependent on the temperature of the components and the reaction continues until one of the reagents is depleted. Chemical sensitisation can be problematic in cold environments, sometimes necessitating additional acidification, in order to achieve the desirable rate of the sensitisation reaction [21].

The reaction underlying chemical sensitisation, i.e., the reaction of nitrite anions and ammonia cations, exhibits relatively slow kinetics. Such kinetics translate into the properties of the EE being strongly dependent on the time elapsed between the sensitisation and initiation (“sleep time”) of the EE charge. A strong dependence of properties on time is sub-optimal, as in order to achieve the planned performance of the EE, a precise timing would be required. Instead, it is more favourable for the majority of changes in the properties of the EE to take place nigh-immediately upon sensitisation and to be followed by a period of noticeably lesser changes.

Due to the above, methods of improving the kinetics of the sensitisation reaction were investigated, resulting in the introduction of thiourea as a substance reacting more readily with nitrite anions than the ammonia cations present in the system [22]. The kinetics of the sensitisation reaction can also be augmented with the use of calcium and strontium cations. These additives have also been found to promote the nitrite-ammonium reaction [23]. Although no mechanism underlying this process was proposed, the introduction of such cations was found to increase the rate of sensitisation, generate smaller and more uniformly dispersed gas bubbles and increase the stability of the sensitised EE.

More recent work on the subject of chemical sensitisation has been dedicated to supplementing the fuel phase with cocoa fat and stearic acid [24]. Supposedly, this addition stabilises the sensitisation process, resulting in an EE exhibiting a homogeneous distribution of nitrogen gas bubbles that is stable for no less than 96 h. Although no material evidence has been given to support these claims, the postulated additives are used in significant amounts and in light of them being noticeably more expensive than the other components of EEs, there may be little economic justification for including them in the EE formulation.

An alternative route to chemically sensitising EEs relies on a reaction between ammonium nitrate and hypochlorites [25]. Although the reaction is described as less hazardous than chemical sensitising with the use of thiourea, this may be debatable, as the reaction yields chloramine as an intermediate product, which may be transformed into highly toxic hydrazine, depending on the reaction conditions [26]. Another potential issue of this invention is that the rate of sensitisation is difficult to control, even with the use of a variety of surfactants, due to the high reactivity of the reagents.

Although much less prominent than the composition of post-detonation gases and threats arising therefrom, the emission of hazardous gases from boreholes containing EE undergoing sensitisation is also a significant risk to personnel. In the case of the nitrite-ammonium reaction, both the inert nitrogen and the hazardous nitric oxide (NO) are produced. It has recently been found that the emission of the latter can be mitigated with the use of nitrosoaromatic sulfonates, such as 5-dimethyl-4-nitrosobenzene sulfonate [27]. Even an addition of 1% wt. of this compound was found to reduce NO emissions from the EE by approx. 70%. That said, it is unclear how the inclusion of such a compound and the retention of NO within the EE may affect its energetic performance or the composition of post-detonation gases, making the usefulness of this novel approach an open question.

## 4. Additives to EEs

Emulsion explosives (EEs) typically exhibit moderately high energetic parameters, such as an ability to perform mechanical work on the order of 80–85% of that exhibited by RDX [8] and a velocity of detonation on the order of up to 4000–4500 m/s. While these parameters are adequate for most civilian applications, they may be insufficient for blasting operations in hard rocks, where the use of nitroester-based energetic materials is prevalent. In order to allow EEs to compete against traditional energetic materials in such areas of applications, it is necessary to improve their energetic performance. The most common approach to augmenting the energetic parameters of EEs involves supplementing them with a variety of additives.

The most commonly employed additives are metal powders, particularly low density metals, such as aluminium and titanium, however more sophisticated as well as simple inorganic compounds of metals, such as their hydrides. The exothermic combustion of metals not only increases the detonation temperature and the positive blast phase duration, but also helps reduce the amount of nitrogen oxides in the post-detonation gases, via promoting dissociation and disintegration of their precursor radicals [28]. The addition of aluminium powder to bulk EE appears to promote its sensitisation, as increasing the Al content in the formulation has lead to decreased EE density 60 min after sensitisation [8]. Although this lowered EE density translates into lower detonation velocity, the overall explosive strength and Trauzl expansion parameters are also increased by approx. 5% and 17% respectively, when compared to the formulation that was not supplemented with Al. Supplementing EEs with titanium powder has a similar effect, as the addition of Ti was found [29] to result in increased brisance, explosion impulse and shock wave energy. In contrast to the use of Al and boron as additives, Ti has the advantage of having a less adverse impact on the thermal stability of the formulation.

**Figure 2:**
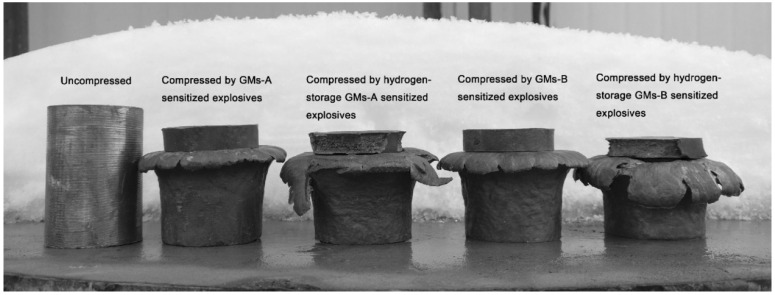
Hess test (lead column compression) results for EEs sensitised with various types of microspheres. Reprinted with the permission of Wiley from [30]. Copyright 2018.

An interesting and environmentally-friendly modification to physically sensitised EEs is to replace hollow or air-filled microballoons with ones filled with hydrogen [30]. Two types of microballoons were studied, with hydrogen filling slightly increasing brisance, detonation velocity and shock impulse (Figure 2). A similar approach to introducing hydrogen into EE formulations relies not on including it within microballoons, but chemically bound, in the form of titanium hydride, which appears to combine the advantages of supplementing EEs with titanium with those achieved by supplementing them with hydrogen [31]. The supplementation resulted in a significant improvement in explosion strength and detonation velocity (increased Trauzl test result by 30% and detonation velocity by 3% in comparison with the unsupplemented EE formulation). The performance was found to also be improved in comparison with a formulation supplemented only with Ti powder, possibly indicating a new direction for EE formulation optimisations.

Modifying the bulk EE sensitising agent solution intended to fine-tune the changes taking place during sensitisation is a novel approach [32], bringing about an improvement to the energetic parameters achievable for EEs. An important advantage of this approach is that it does not require any modification of the EE matrix formulation, making it compatible with existing EE manufacturing and loading solutions. In terms of EE performance, this approach was found to afford increased brisance (by up to 32%) and detonation velocity (by up to 19%), while limiting the emission of carbon monoxide during detonation, compared to a standard commercial EE formulation. An added value of this approach is an improvement to the kinetics of sensitising process, with the density of the modified formulations decreasing more rapidly than the commercial EE formulations and maintaining a more stable density after this initial change, making for a more user-friendly explosive for blasting operations.

## 5. Energetic Properties of EEs

### 5.1. Velocity of Detonation

Velocity of detonation (VoD) is among the parameters that are most commonly used for describing the performance of energetic materials. It is the maximum velocity at which a detonation wave can propagate in a given energetic material. The VoD value is a function of both the physicochemical properties of the energetic material formulation (e.g., chemical composition, density) and the features of the investigated charge (e.g., charge diameter).

**Figure 3:**
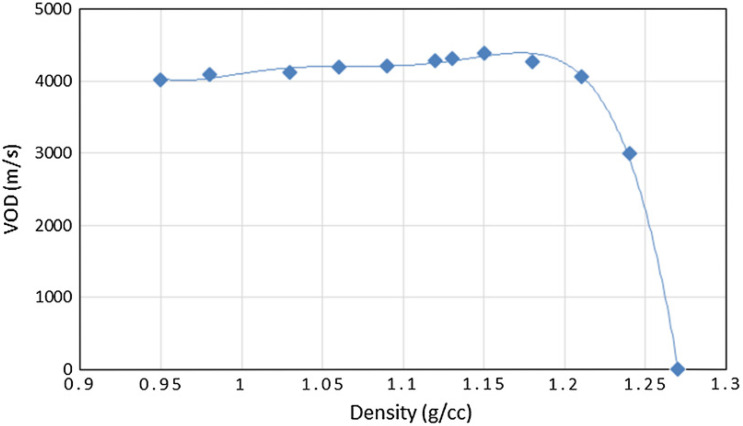
Dependence of VoD on the density of a chemically sensitised EE. Reprinted with permission of Springer Nature from [33]. Copyright 2017.

Of the aforementioned parameters, the influence of density on the VoD of EEs has been studied in greatest detail. In general, the VoD of EEs increases with density to a certain critical point, at which a maximum VoD value is observed. Increasing EE density beyond this results in a rapid decrease of VoD, followed by the inability of the EE to undergo detonation (Figure 3). It should be noted, however, that the measured density of the EE is a function of both the population of pores and their size distribution, therefore the same charge density may result from different combinations of these two parameters. Consequently, the shape of the VoD vs density curve may vary, depending on the sensitisation method (with physical sensitisation being expected to afford higher pore size distribution repeatability than chemical sensitisation methods).

EEs are considered to exhibit a relatively high contact between the oxidising agent and the fuel, due to their highly dispersed phase volume concentration, exceeding the limit achieved for suspensions. This property allows excellent detonation parameters to be obtained for EEs, unlike what is seen for the more traditional energetic materials utilising ammonoium nitrate (e.g., ANFO). The differences can be seen mainly in the velocity of detonation. While traditional ANFO-type materials can barely achieve a VoD on the order of 3000 m/s, EEs typically achieve VoD values in excess of 4500 m/s.

In the case of cartridged EE, the velocity of detonation is constant over time, as physical sensitisation is typically highly stable, due to the use of microballoons. Despite being stable over time, cartridged EEs are susceptible to elevated temperature, a fairly frequent circumstance in blasting operations, with rock strata temperatures in underground mines often having a temperature in excess of 40–50 °C. At such temperatures, the EE is partially or completely liquefied, which significantly changes its performance. Simultaneously, elevated temperature can promote phase separation phenomena, particularly crystallisation of ammonium nitrate. It has been found that conditioning cartridged EEs at elevated temperatures even for 2–3 h was sufficient to result in a noticeable decline in VoD in regards to the initial VoD value [34].

In contrast to cartridged EEs, the use of chemical sensitising for bulk EEs offers lower stability, as the reaction underlying the sensitisation of the EE cannot be stopped at will. Consequently, for bulk EEs, the time elapsed between sensitising the EE matrix loaded into a borehole and initiating the charge, which is often referred to as “sleep time”, results in changes in the density and VoD of the EE. Moreover, the magnitude and pace of the change in the properties of the EE during the sleep time is dependent on environmental factors, primarily temperature [34].

An interesting observation is that during the gradual decrease in bulk EE density, the VoD does not continuously decrease but initially increases, likely due to the growing population of hot spots within the EE. The sleep time interval and EE density corresponding to peak VoD are not constant, even for a single EE formulation, as changing the speed of the pump used for loading the EE into the borehole was found to influence the occurrence of this VoD peak, likely due to achieving different degrees of mixing between the EE matrix and sensitising agent [35].

In another study, for an EE at room temperature, a sleep time of 15 days was found to result in VoD declining by approximately 10%, with a greater decline being observed for EE in boreholes than for unconfined EE samples [36] (Table 1).

Such changes to the VoD value of the EE formulation are highly undesirable, particularly when blasting is conducted under a variety of external conditions, as the rates of EE sensitisation and, therefore, the performance of the energetic material will vary significantly.

An obvious question, related to the issue of loss of EE energetic performance during sleep time, is that of the limit of this loss in the long term. Although the loss of performance, including the decline of VoD, was found to gradually decelerate, it has been observed to continue taking place even after 6 months have elapsed since loading the sensitised EE into plastic tubes [37]. Interestingly, even though the commercially available Emulinit 8L EE required the use of a booster charge to be initiated after 6 months, it still achieved approx. 70% of the original VoD value.

It is important to note that multiple factors can simultaneously affect the performance of EEs. It has been recently found that, despite the density and viscosity of a EE formulation being monitored, the velocity of detonation (VoD) was found to vary significantly for charges in boreholes across different blasting sites [38]. The occurrence of such changes were attributed to differing external conditions: the presence or lack of water in the boreholes, the hardness and brittleness of the rock strata and the presence of cracks and voids within these strata.

An interesting approach to optimising the performance of EEs in softer rocks is to employ air gaps within the explosive column loaded into the boreholes. It has been shown that with careful choice of the dimensions and distribution of such air gaps, the velocity of detonation is only slightly reduced, while allowing a noticeable reduction in the amount of EE loaded into the borehole [39]. That said, no information was presented as to whether the amount of mechanical work (i.e., volume of rock mined and the degree of fragmentation in the mined rock) has been adversely affected by the introduction of those air gaps and, if so, what the magnitude of the decline of the performance is.

The concept of critical diameter, that is, the lowest diameter of an energetic material charge that allows detonation to be sustained along its length, is well-known for energetic materials, including EEs [40]. Increasing the charge diameter above the critical diameter, however, can lead to increasing the performance of non-ideal energetic materials, such as ammonium nitrate-based energetic materials and EEs. In the case of EEs, it was shown that their VoD is strongly dependent on charge diameter, particularly when the charge diameter is only slightly larger than the critical diameter for that EE formulation (Table 2) [41].

### 5.2. Post-Detonation Gases

Energetic decomposition of EEs yields significant amounts of gaseous products, i.e., water vapour, carbon monoxide, carbon dioxide, nitrogen and nitrogen oxides. Among those, the emission of carbon monoxide and nitrogen oxides is seen as problematic, particularly in the case of underground blasting operations, due to the toxicity of those gases. The composition of post-detonation gases is a function of both the composition of the EE (as the oxidising agent to fuel ratio influences the carbon monoxide to carbon dioxide and nitrogen to nitrogen oxides ratios) as well as external circumstances (as cracks in the walls of the borehole or the presence of water within can lead to the occurrence of incomplete detonation) [39].

The composition of post-detonation gases can be altered by supplementing the EE formulation with additives intended to modify the energetic decomposition process. Such additives tend to be extremely varied in the existing literature, as they can fulfil the role of auxiliary oxidising agent, auxiliary fuel or act as catalysts.

The addition of aluminium and of ammonium nitrate to an EE formulation has recently been investigated in the context of their impact on the composition of post-detonation gases [42]. The interpretation of the results of this work, however, can be considered controversial, as the composition of the EE formulation was heavily modified to achieve a constant oxygen balance and those changes were not taken into account when reporting the amounts of post-detonation gases produced. This leads to obviously flawed conclusions, such as that supplementing the EE with an oxidising agent (ammonium nitrate prills) leads to an increased emission of carbon monoxide by a factor of 1.6–1.9, even though the ammonium-nitrate supplemented formulation contains a much greater share of carbon-based fuel than the initial EE formulation.

More recent research shows results contrary to the above, as supplementing the EE with a combination of ammonium nitrate and sodium perchlorate was found to decrease the amounts of carbon monoxide and nitrogen oxide present in the post-detonation gases (Table 3) [32]. Although supplementing EEs with oxidising agents did not resolve the issue of NO_2_ emission, the lowered CO emission significantly decreases the risk associated with utilising such modified EE formulations in underground blasting operations.

In order to provide context for the above, it should be noted that exposure to 50 ppm of CO for approximately 30 min is considered non-threatening, whereas exposure to 200 ppm of CO concentration induces the first symptoms of carbon monoxide poisoning [43]. Although their toxicity is not as acute as in the case of CO, both nitric oxide (NO) and nitrogen dioxide (NO_2_) are also highly toxic and corrosive gases [44].

Predicting the properties of energetic materials based on their composition is an extremely difficult but worthwhile task, as it helps to minimise the exposure of personnel to those materials by reducing the number of experiments required to determine and fine-tune the properties of energetic materials being developed. Recently, a model for predicting the amount of post-detonation gases produced upon the detonation of ANFO- and EE-type energetic materials has been proposed [45]. Despite being an early model, an acceptable match to data reported in the literature was achieved.

## 6. Applications of EEs

EEs are commonly utilised in civilian blasting operations, particularly in mining. In this application, the use of bulk EEs is continuously increasing its market share (Figure 1) due to the increased safety parameters of those materials, as well as due to the ability to minimise the exposure of personnel to the threat of explosion than in the case of using traditional energetic materials [46].

The use of EEs in blasting operations associated with mining often encounters practical issues arising from the conditions existing in boreholes, both natural (e.g., temperature of the rock) and produced during the drilling of boreholes (e.g., presence of rock fragments in the borehole, cracking of borehole walls). In this regard, both the loading of boreholes and the reliability of detonation taking place across the entire length of the borehole are significant and common issues. The issue of loading boreholes is largely technical and requires careful control over the pressure and flow rate of the loaded EE.

Conversely, the reliability of detonation may easily be compromised, particularly in deep boreholes, due to the hydrostatic pressure exerted by the pillar of the EE. Depending on borehole depth, this pressure can be large enough to induce gas bubble compression in the bottom section of the borehole. This can be avoided by limiting borehole depth or resolved to some extent, by utilising a higher initiating stimulus. Another phenomenon that needs to be taken into account in regards to the reliability of detonation is the timing of detonations in neighbouring boreholes. When the blast wave caused by nearby detonation travels through a borehole containing EE, momentary compression of the gas bubbles present in the EE takes place, causing it to temporarily lose sensitivity to initiation. If this issue is not taken into account during the planning of blasting operations, it can even lead to misfires.

The use of EEs for testing the mechanical properties of construction materials is a non-obvious application that is of particular importance, both due to the relevance of data that can be acquired and due to the ever-present threat of bomb terrorism. In a recent work, the experimental testing of reinforced concrete was combined with a theoretical work-up, in order to yield a methodology for testing and modelling the fracture and resistance of brittle materials to blast loads [47]. In the given experimental case, the model was used to explain the role of the reinforcing material in limiting the propagation of cracks in the bulk of the concrete, but other types of reinforced concrete materials have also been recently studied [48], providing an interesting insight for developing blast-resistant constructions. In this aspect, EEs have also been used to investigate the ability of pipelines to withstand blast loads [49].

**Figure 4:**
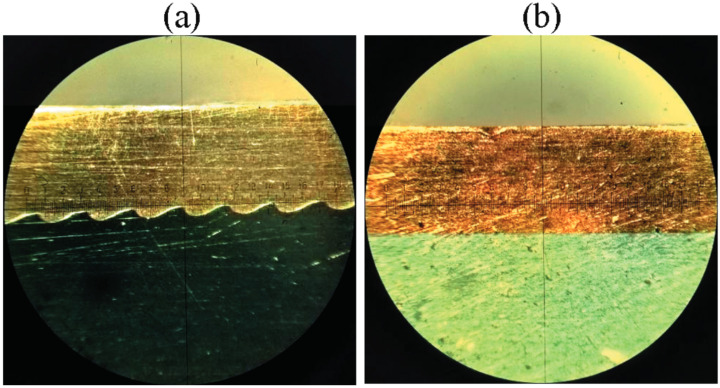
Cross-sections of (**a**) copper-steel and (**b**) copper-aluminium bonding zones produced via explosive welding with the use of EEs. Reprinted with the permission of Springer Nature from [51]. Copyright 2018.

Another non-straightforward application of EEs is in explosive welding. Compared to traditional welding methods, explosive welding allows a broader range of metals and alloys to be combined. In this approach, the use of EEs instead of traditional, high-performance energetic materials was found to allow welding even highly dissimilar materials (Figure 4), due to the lesser strain induced by the detonation of EEs [50,51], enabling the fabrication of a broader variety of composite materials.

The issue of water bodies, through which marine transport takes place, being covered with ice is important from an economical standpoint, particularly so in sub-polar and polar locations. One of the methods of removing such ice coverage is to employ energetic materials, due to their significant brisance. In the reported case, EEs were used for this purpose and a model of the behaviour of ice under blast loading was developed, potentially opening up a new avenue of application for these energetic materials [52].

Powder metallurgy is a relatively young field, dealing with the processing of metals from powders. In this field, energetic materials are frequently used as sources of mechanical work for compressing the processed powders into solid elements. The use of EEs for this purpose has been recently reported and even though metal powder solidification was readily achieved, the amount of used EE is relatively large, in the range of 500–1000 g for compressing approx. 60 g batches of iron powder [53]. Even though the method appears to be inefficient, it was found that the amount of EE used can be altered in order to tailor the hardness of the resultant solid elements, which is of some practical significance.

### Safety Considerations

An important risk associated with conducting blasting operations is the occurrence of ground vibrations, often referred to as para-seismic oscillations. Such vibrations can propagate over long distances, damage and even topple buildings. Recently, a dynamic finite element model has been utilised to model these vibrations [54]. Although the proposed model predicted higher vibration magnitude than was experimentally observed, the predictions were fairly accurate over shorter distances. The observed inaccuracies were primarily attributed to the model not taking into consideration the occurrence of various rock strata across the vibration propagation distance [55]. Even though the model needs to be refined significantly, it is a promising step forward in terms of predicting risks associated with conducting blasting operations.

An important consideration in terms of EE safety is their possible contamination. Among commonly encountered contaminants, Fe^2+^ ions have been found to lower the thermal decomposition temperature of EEs from approximately 280 °C to approximately 271 °C and to promote crystallisation in EE matrices [56].

## 7. Conclusions

Despite high entry requirements into the subject of EEs, the development of this class of energetic materials continues and appears to be attracting increasing research interest over recent years. This is due to both the significant improvements to the performance and the safety features of these materials, making them an increasingly more favourable alternative for traditional energetic materials in general and for nitroester-based energetic materials in particular.

The issues of the rheology and stability of both the EE matrices and the sensitised EEs have been a particular focus among recent works due to the practical considerations associated with the instruments and processes of sensitising and loading the EEs into boreholes. This aspect of EE development is also more readily available, as testing the rheology of even sensitised EEs does not necessitate conducting blasting operations and can be safely conducted in a specialised laboratory setting.

It should be noted, however, that the rheology of EEs ties in with the issue of the population and size distribution of pores in the sensitised EE. Despite multiple studies, the subject remains controversial and requires further experimental exploration. A particularly important question in this regard is that of the nature of the impact of the porosity parameters on the energetic performance and sensitivity of the EEs.

In contrast, the works dedicated to improving the energetic performance of EEs are significantly fewer, due to the extremely significant need to perform blasting repeatedly in a variety of experimental configurations, while ensuring the safety of both personnel and the utilised instruments. Even so, such works are highly valuable, both scientifically—pushing forward our understanding of the processes occurring in those energetic materials and the myriad factors influencing these processes—as well as economically, due to the fact that even a minute improvement in the performance of EEs translates into more efficient blasting operations, allowing more rock to be mined with a lesser total amount of energetic material utilised.

Among the above, particular attention should be paid to emerging additives to EE formulations, as the inclusion of even relatively common substances as additives was proven to significantly improve various properties of the EEs. This trend is expected to bring even further improvements to the properties and performance of EEs in the future, helping promote new, higher standards in the development and use of energetic materials.

The implementation of increasing standards for EEs inevitably involves discussion of the personnel health and environmental impacts of such materials. In this regard, the composition and amount of gases produced during the energetic decomposition of EEs is an essential, but under-explored issue. Although some strides have been made in limiting the emissions for EEs, the subject remains a significant issue, as current EE formulations produce carbon monoxide and nitrogen oxides in the post-detonation gases. Reducing these emissions remains an important, unresolved issue that needs to be overcome, in order to achieve “green” EEs.

## Figures and Tables

**Table 1:** Effect of sleep time on the VoD of EEs [36,37].

Density [kg/m^3^]	Sleep Time	VoD [m/s]
**Unconfined EE **		
1120	0 days	4130
1100	3 days	4200
1090	6 days	4100
1110	9 days	4000
1120	12 days	3840
1100	15 days	3780
**EE in Borehole**		
1120	1 day	4920
1130	7 days	4706
1160	15 days	4359
**EE in Plastic Tubes**		
-	30 min	4230 ± 105
-	60 min	4005 ± 40
-	180 min	3732 ± 20
-	24 h	3543 ± 27.5
-	48 h min	3420 ± 32.5
-	7 days	3330 ± 40
-	14 days	3153 ± 25
-	31 days	3100 ± 12.5
-	4 months	3017 ± 15
-	6 months	2930 ± 25

**Table 2:** Effect of charge diameter on the VoD of EEs [41]. Average VoD (n = 3) values are reported.

Charge Diameter [mm]	VoD [m/s]
**Emulinit 7L**	
32	-
40	3700 ± 40
50	3910 ± 30
**Emulinit 8L**	
32	3310 ± 170
40	3630 ± 30
50	3990 ± 55

**Table 3:** Summary of the average composition of post-detonation gases for a commercial EE (Emulinit 8L) and EEs supplemented with additional oxidising agents (BK-1, BK-2). Reprinted from [32] under a CC BY license.

Emulinit 8L	CO_2_	CO	NO_2_	NO
Concentration [ppm]	4583 ± 45	162 ± 11	1.4 ± 0.2	20.0 ± 7.4
Unit mass emission [dm^3^/kg]	114.8 ± 1.1	4.11 ± 0.28	0.04 ± 0.01	0.51 ± 0.19
**BK-1**	**CO_2_**	**CO**	**NO_2_**	**NO**
Concentration [ppm]	4664 ± 6	100 ± 4	1.5 ± 0.2	11.6 ± 2.8
Unit mass emission [dm^3^/kg]	117.1 ± 0.9	2.51 ± 0.12	0.04 ± 0.01	0.29 ± 0.07
**BK-2**	**CO_2_**	**CO**	**NO_2_**	**NO**
Concentration [ppm]	4553 ± 24	136 ± 18	1.2 ± 0.2	11.0 ± 5.3
Unit mass emission [dm^3^/kg]	115.3 ± 0.4	3.45 ± 0.46	0.03 ± 0.01	0.28 ± 0.13
